# Red Light Is Effective in Reducing Nitrate Concentration in Rocket by Increasing Nitrate Reductase Activity, and Contributes to Increased Total Glucosinolates Content

**DOI:** 10.3389/fpls.2020.00604

**Published:** 2020-05-14

**Authors:** Angelo Signore, Luke Bell, Pietro Santamaria, Carol Wagstaff, Marie-Christine Van Labeke

**Affiliations:** ^1^Department of Agricultural and Environmental Science, University of Bari Aldo Moro, Bari, Italy; ^2^School of Agriculture, Policy and Development, University of Reading, Reading, United Kingdom; ^3^Department of Food and Nutritional Sciences, University of Reading, Reading, United Kingdom; ^4^Department of Plants and Crops, Faculty of Bioscience Engineering, Ghent University, Ghent, Belgium

**Keywords:** *Eruca sativa*, *Diplotaxis tenuifolia*, soilless culture, glucosinolates, light emitting diodes

## Abstract

Rocket cultivation is increasing to supply the expanding ready-to-eat market because of its unique taste, but crops are often over fertilized to avoid nitrogen deficiencies. This leads to nitrate accumulation in leaves, and the products of their degradation (nitrites and nitrosamines) have been related to several health problems. Nitrate concentrations in rocket and other leafy vegetables are subject to limits by the EU legislation, yet rocket holds a great nutritional value. Degradation products of glucosinolates (isothiocyanates) have been consistently linked with benefits to human health. We investigated the influence of nitrogen application (1 and 8 mM), species [*Eruca sativa* (L.) Cav. and *Diplotaxis tenuifolia* (L.) DC.] and light spectrum (full spectrum, red, blue and red + blue) on the nitrate concentration, nitrate reductase activity and glucosinolate content of rocket grown in a soil-less system. Red light decreased the nitrate concentration with respect to the blue spectrum (4,270 vs. 7,100 mg⋅kg^–1^ of fresh weight, respectively), but such reduction was influenced by the species and the nitrogen level (significantly higher in *D. tenuifolia* and with the higher concentration of N). The nitrate reductase activity increased under red light in *D. tenuifolia*, with the lower N concentration. Rocket is known to contain several health-promoting compounds mainly antioxidants and glucosinolates, as secondary metabolites that act as part of plant defense mechanisms. The total content of glucosinolates was mainly affected by the species (*D. tenuifolia* showed the highest concentrations). Our results will help growers to tailor light spectra with the aim of reducing nitrate concentration and to remain within EU legislative limits, without any detrimental influence on other qualitative parameters in rocket.

## Introduction

The name “rocket” (or rucola, arugula, and roquette) is a collective term to indicate several species of the Brassicaceae family, indigenous to the Mediterranean region, whose leaves are characterized by a pungent taste, that are used to flavor salads. Four species are used for human consumption, namely *Eruca sativa* (synonym *Eruca vesicaria;* salad or cultivated rocket), *Diplotaxis tenuifolia* (wild or perennial rocket), *Diplotaxis muralis* and *Diplotaxis erucoides*, but only the former two are cultivated on a large scale (Bianco et al., 1998; Bell and Wagstaff, 2014). Leaves are typically sold in salad bags (mixed or alone) and their commercial importance has increased significantly across the globe. Indeed, even though rocket has been consumed since ancient times (Hall et al., 2012), it was still considered as an underutilized species until 20 years ago (Bianco, 1995). As with many Brassicaceae species, rocket has a high concentration of compounds with beneficial aspects for human health, such as glucosinolates (GSLs), flavonols and vitamin C. For such a crop, there are also reports that leaves have diuretic and anti-inflammatory properties, as well as beneficial cardiovascular effects (Mahran et al., 1991; De Feo and Senatore, 1993; D’Antuono et al., 2008; Egea-Gilabert et al., 2009; Björkman et al., 2011).

Rocket species are known to be a hyper-accumulators of nitrate (Santamaria et al., 1998a). Nitrate is considered harmful for human health when present in high concentrations, as an anti-nutritional dietary component. Through its reduction to nitrite and conversion to nitrosamines *in vivo*, it has been linked with methemoglobinemia and cancer (Santamaria, 2006). Nitrate has been linked also with positive health aspects, such as cardioprotective properties (Bondonno et al., 2016). The picture relating to its health effects is therefore subject to much debate.

The ability of rocket to hyper-accumulate nitrate, and in particular in *D. tenuifolia* (Santamaria et al., 2002), is so high that the acceptable daily intake (ADI) could be exceeded by eating less than 50 g rocket with a median nitrate concentration (European Food Safety Authority, 2008). In recent years, the harmfulness of nitrate has been disputed, and there are some studies that have proposed that nitrate has to be considered as a nutrient necessary for health, rather than as a contaminant which needs to be restricted. Such studies have debated, for example, the function of nitrate with respect to blood pressure and cardiovascular health (Bondonno et al., 2016; Jonvic et al., 2016; Ashworth and Bescos, 2017), and the significant reduction of oxygen consumption and higher total muscle work during moderate-intensity exercise (Porcelli et al., 2016).

Despite the lack of scientific consensus, the European Commission [with the Regulation (EC) N. 1258/2011, European Commission, 2011; amending Regulation (EC) N. 1881/2006] has arbitrarily set maximum levels for nitrates (NO_3_^–^) in foodstuffs, including rocket (for which the limits are 6,000 and 7,000 mg NO_3_^–^⋅kg^–1^, if harvested between 01 April to 30 September and from 01 October to 31 March, respectively). These limits cannot be legally exceeded, and product in breach of the regulations cannot be sold in the EU.

The factors influencing nitrate accumulation in vegetables are numerous; for example the species, the organ that is consumed (with leaves and petioles playing a major role), the fertilization and light intensity (Santamaria, 2006; Weightman et al., 2012). Among the above factors, the most important ones are nitrogen availability and light intensity. With regard to nitrogen fertilization, there are some strategies that can be used for reducing the nitrate content; in particular in hydroponic systems (Santamaria et al., 1998a, b).

It has been demonstrated that nitrate accumulation is higher with low light intensity (Premuzic et al., 2002). This could lead to major problems, particularly in northern European countries, in which the light intensity is usually lower than the southern countries. But even in the latter ones, if dull conditions and high temperatures persist the days before the harvest, nitrate concentrations can be increased (Weightman et al., 2012). Under these conditions nitrate becomes an important anion osmoticum due to a lower carbon source flux (Seginer, 2003); but also nitrate reductase activity influences the nitrogen flux (NR; the enzyme converting nitrate to nitrite prior to its assimilation into ammonium, amino acids and, as latest step, proteins) as it is activated by light (Weightman et al., 2012). Low light intensity may therefore induce a higher nitrate content (Santamaria, 2006).

With respect to light, greenhouse growers can influence light intensity through supplementary lighting, while such technique is hardly applied in soil-bound production. Moreover, the use of LED lights is increasing in rocket production as it allows cultivation in closed or semi-closed environments, and an ability to modify plant secondary metabolism (Ouzounis et al., 2015) to favor the accumulation of substances useful for human health. However, the influence of light spectra is various, and depends on several parameters. For example, Bian et al. (2018) underlined the importance of green light on nitrate reduction in lettuce, while others (Viršilë et al., 2019) found that a mix of red and blue light decreased nitrate but increased nitrite concentrations on tatsoi. In addition, the effect of light spectra on nitrate concentration has been demonstrated to be genotype dependant (Viršilë et al., 2020).

Given the above premises, the scope of our work was to compare the effects of four light treatments (Full Spectrum, FS; BLUE, RED, and RED + BLUE) on two species of rocket grown with two levels of nitrogen (N – 1 and 8 mmol⋅L^–1^) in nutrient solution (NS), and their effects on NR activity, yield, nitrate concentration and GSLs in the leaves.

## Materials and Methods

A trial was carried out in controlled environment growth chambers located at the Department of Plant and Crops, Ghent University (Belgium).

### Plant Material and Experimental Setup

Seeds of rocket (*E. sativa* and *D. tenuifolia*) were sown into rockwool plugs (diameter 2 cm, height 2.7 cm – Grodan BV, Netherlands) soaked with tap water, which electric conductivity was 0.5 dS⋅m^–1^, and arranged into polystyrene trays. The sowing was done on January 29 and the entire cycle (from sowing to harvest) lasted 66 days. Subsequently, the rockwool plugs were covered with wetted vermiculite and the trays were covered with a transparent plastic film, to avoid humidity loss until complete germination. Germination took place in a dark chamber (temperature: 20°C) for 3 days. Thereafter, the plastic film was removed and the trays were moved onto an aluminum bench, under artificial light (high pressure sodium lamps – SON-T, 400 W, Philips, Eindhoven, Netherlands) with a light intensity (at canopy level) of 200 μmol⋅m^–2^⋅s^–1^ and a photoperiod of 14/10 h (day/night). The plants were irrigated with tap water, four times a day, through an ebb-and-flood system.

Four days after the germination, the fertigation started (see section “Nutrient Solution” for the NS composition and management). The NS concentration was 1/4 of the final concentration for the first 10 days, and then 1/2 of the final concentration for the following 4 days. Subsequently the plants were transplanted, at the second true leaf stage, into polystyrene boxes, whose internal measures were 23, 27, and 11 cm for length, width, and height, respectively, which available volume, after a waterproof coating with plastic, was equal to 5.5 L, containing the NS (nutrient composition reported in Table 1). Each box held five rockwool plugs, and each plug contained four and eight plants for *E. sativa* and *D. tenuifolia*, respectively. Twenty-one days after the transplant, the treatments were differentiated with respect to the nitrogen concentration and the light spectrum. The experimental design used was a split-plot design over sites, with the main factor (sites) represented by the light treatment. In every light treatment, corresponding to one bench containing the boxes, the nitrogen concentration was the main plot and species were the subplot factor. Every treatment was replicated three times.

**TABLE 1:** Levels of the macro and microelements into the nutrient solutions.

Macroelement	N level (mM)	
	1	8

	**Element concentration (mM)**	

N	1.00	8.00
K	4.37	4.39
P	1.32	1.32
Mg	1.22	1.22
Ca	2.64	4.65
S	2.37	0.90

**Microelement**	**Element concentration (μM)**	

Fe	20	
Cu	0.5	
Zn	2	
Mo	0.1	
Mn	5	
B	25	

### Nutrient Solution

The NS was prepared by using lab salts (purity >99%), with the exception of iron, for which Sequestrene 138 Fe 100 SG (Syngenta, Oosterzele, Belgium) was used. The NS used differed for nitrogen concentration (1 or 8 mmol – Table 1). Every day, if needed, fresh NS was added to the boxes. The pH value of the NS was maintained in the range 6–7 by adding HCl (1 M; as required) while the EC value were 1.20 and 1.65 dS⋅m^–1^ for the nitrogen concentration of 1 and 8 mM, respectively. The oxygenation of the NS was provided continuously by a compressor connected to plastic pipes that were submerged into the NS.

### Light Treatments

Four light treatments were set up, namely: FS (full spectrum by the means of a solid state plasma light – Gavita Holland, Aalsmeer, Netherlands), blue (BLUE, peak at 460 nm – GreenPower LED research module, Philips, Eindhoven, Netherlands), red (RED, peak at 660 nm – GreenPower LED production module), as well as a combination of red with blue (R + B, 75% red + 25% blue) with a programmable LED experimentation system (CI-800, CID Bio-Science, Camas, WA, United States), respectively. Light intensity was 150 μmol m^–2^ s^–1^ at canopy level, which results in a daily light integral of 7.5 mol m^–2^.

### Harvesting and Sampling

The harvest was performed manually by cutting the plants with scissors (1 cm above the box level) and by immediately weighing the material to determine the fresh weight. Of the fresh material, a quota of stems and leaves (a balanced mix of apical, median and basal zone) was immediately ground in liquid nitrogen (IKA^®^ A11 Basic Analytical Mill, Germany) and the resulting powder was stored at −80°C for the NR assay and for the determination of GSL content. Subsequently, a quota of the fresh weight (around 20 g) for every replication was placed into a ventilated oven at 65°C, until its weight was constant, for the dry matter determination. Afterward, material was ground to a powder for the analysis of nitrates in the tissues.

### Analytical Determinations

#### Leaves NR Assay

Leaves (1 g) were ground in a chilled mortar with 2 mL of extraction buffer as described previously by Reda (2015). The crude supernatant was used for the measurement of NR in the absence of MgCl_2_ (total activity – NR_total_) and/or in the presence of MgCl_2_ (actual activity – NR_act_).

The reaction medium contained 50 mM Hepes-KOH, pH 7.5, 5 mM EDTA (NR_total_) or 5 mM MgCl_2_ (NR_act_), 10 mM KNO_3_ and crude extract. Following the addition of 0.2 mM NADH, the reaction was carried out in a heated water bath at 27°C for 5 min and then stopped with 0.066 mL of 1 mM zinc acetate. The mixture was centrifuged (18,000 *g* for 18 min at 4°C) and the nitrite concentration was determined colorimetrically at 540 nm. The activation state of NR was calculated as the ratio NR_act_/NR_total_ activity expressed as a percentage.

#### Nitrate Analysis

Nitrate concentrations were determined by ion chromatography (Dionex model DX500; Dionex Corporation, Sunnyvale, CA, United States) with a conductivity detector, using the pre-column IonPack AG14 and the column of separation IonPack AS14 (Signore et al., 2008). Ultrapure water at 18 MΩ/cm (Milli-Q Academic Millipore) was used in all the analysis.

#### Glucosinolate Extraction and Analysis

##### Reagents and chemicals

All solvents and chemicals used were of LC–MS grade and obtained from Sigma-Aldrich (Poole, United Kingdom) unless otherwise stated.

##### Glucosinolate extraction

The extraction protocol used was taken from Bell et al. (2015). Briefly, two experimental replicates of each biological rep (*n* = 6) were prepared as follows: 40 mg of ground powder was heated in a dry-block at 75°C for 5 min; 1 mL of preheated 70% (v/v) methanol (70°C) was then added to each sample and placed in a water bath for 20 min at 70°C. Samples were centrifuged for 5 min (12,000 rpm, 20°C) to collect loose material into a pellet. The supernatant was then filtered using 0.22 μm Acrodisc syringe filters with Supor membrane (hydrophilic polyethersulfone; VWR, Lutterworth, United Kingdom) into fresh Eppendorf tubes. Samples were frozen at −80°C until analysis by LC–MS.

##### LC-MS analysis

Immediately before LC–MS analysis, each sample was diluted with 9 mL of HPLC-grade water. Samples and standards were run in a random order with QC samples (Dunn et al., 2012). An external standard of sinigrin hydrate was prepared for quantification of GSL compounds. The preparation was as follows: a 12 mM solution was prepared in 70% methanol. A dilution series of concentrations was prepared as an external calibration curve with HPLC-grade water (112, 56, 42, 28, 14, and 5.6 ng⋅μl^–1^; sinigrin correlation coefficient *y* = 28.06; *r*^2^ = 0.999; Jin et al., 2009). Relative response factors (RRFs) were used in the calculation of GSL concentrations where available. Where such data could not be found for intact GSLs, RRFs were assumed to be 1.00.

LC–MS analysis was performed in the negative ion mode on an Agilent 1260 Infinity Series LC system (Stockport, United Kingdom) equipped with a binary pump, degasser, autosampler, column heater, diode array detector, and coupled to an Agilent 6120 Series single quadrupole mass spectrometer. Separation of samples was achieved on a Gemini 3 μm C18 110 Å (150 mm × 4.6 mm) column (with Security Guard column, C18; 4 mm × 3 mm; Phenomenex, Macclesfield, United Kingdom), as recommended by Ares et al. (2014). GSLs were separated during a 40-min chromatographic run, with 5-min post-run sequence. Mobile phases consisted of ammonium formate (0.1%; A) and acetonitrile (B) with the following gradient timetable: (i) 0 min (A–B, 95:5, v/v); (ii) 0–13 min (A–B, 95:5, v/v); (iii) 13–18 min (A–B, 40:60, v/v); (iv) 18–26 min (A–B, 40:60, v/v); 26–30 min (A–B, 95:5, v/v); (v) 30–40 min (A–B, 95:5, v/v). The flow rate was optimized for the system at 0.4 mL/min, with a column temperature of 30°C, and 25 μl of sample was injected into the system. Quantification was conducted at a wavelength of 229 nm (DAD).

MS analysis settings were as follows: API-ES was carried out at atmospheric pressure in negative ion mode (scan range m/z 100–1500 Da). Nebulizer pressure was set at 50 psi, gas-drying temperature at 350°C, and capillary voltage at 2,000 V. Compounds were identified using their primary ion mass and by comparing relative retention times with those published in the literature (Lelario et al., 2012). All data were analyzed using Agilent OpenLAB CDS ChemStation Edition for LC-MS (Agilent, version A.02.10).

### Statistical Analysis

Treatment means were compared using orthogonal contrasts with one degree of freedom (Steel and Torrie, 1988). Three comparisons were made for light treatments: (i) Full light vs. the three light spectra (BLUE, RED, and RED + BLUE LED); (ii) R + B vs. R, B; (iii) R vs. B. Data were subjected to the general linear model procedure (SAS Institute, Cary, NC, United States). The variance results of the main treatments, interactions and contrasts is reported in Table 2.

**TABLE 2:** Summary table of variance of the main treatments and interactions.

Treatment	Y	DM	NC	NRA			GSLs												
				Act	Tot	AcSt	4HGB	4MGB	GBC	DMB	GER	GNPF	GRA	GSV	DGTB	PRO	GAL	GNT	Tot
Species (S)	ns	***	ns	***	**	ns	***	**	***	**	*	**	***	ns	ns	*	ns	ns	**
Nitrogen level (N)	*	**	**	*	*	ns	ns	ns	*	ns	ns	ns	ns	ns	ns	ns	*	ns	ns
Light (L)	ns	ns	***	**	**	ns	ns	ns	ns	ns	ns	ns	ns	ns	ns	ns	ns	ns	ns
FS vs. LED	ns	ns	ns	**	**	ns	*	*	ns	ns	ns	ns	ns	ns	ns	ns	ns	ns	ns
R + B vs. R,B	*	ns	*	ns	ns	ns	ns	ns	ns	ns	ns	ns	ns	ns	*	ns	ns	ns	ns
R vs. B	ns	*	***	**	**	ns	ns	ns	ns	ns	ns	ns	ns	ns	ns	ns	ns	ns	ns
S × N	ns	*	ns	**	**	ns	ns	ns	*	ns	ns	**	**	**	ns	*	ns	ns	*
S × L	ns	ns	ns	***	***	ns	ns	ns	ns	***	ns	ns	ns	*	ns	ns	ns	ns	*
S × (FS vs. LED)	ns	ns	ns	**	**	ns	ns	ns	ns	ns	ns	ns	ns	ns	ns	ns	ns	ns	ns
S × (R + B vs. R,B)	ns	ns	ns	ns	ns	ns	ns	ns	**	***	ns	ns	ns	**	ns	ns	ns	ns	**
S × (R vs. B)	ns	*	ns	***	***	ns	ns	ns	ns	ns	ns	ns	ns	ns	ns	ns	ns	ns	ns
N × L	ns	ns	*	***	**	ns	ns	ns	ns	*	ns	ns	ns	ns	ns	ns	ns	ns	ns
N × (FS vs. LED)	ns	ns	ns	**	*	ns	ns	ns	ns	**	ns	ns	ns	ns	ns	ns	ns	ns	ns
N × (R + B vs. R,B)	ns	ns	*	ns	ns	ns	ns	ns	ns	*	ns	ns	ns	ns	ns	ns	ns	ns	ns
N × (R vs. B)	ns	ns	**	***	***	ns	ns	ns	ns	ns	ns	ns	ns	ns	ns	ns	ns	ns	ns
S × N × L	ns	ns	ns	***	***	ns	ns	ns	ns	ns	ns	ns	ns	*	ns	ns	ns	ns	ns
S × N × (FS vs. LED)	ns	ns	ns	**	**	ns	ns	ns	ns	*	ns	ns	ns	ns	ns	ns	ns	ns	ns
S × N × (R + B vs. R,B)	ns	*	ns	ns	ns	ns	ns	ns	*	ns	ns	ns	ns	*	ns	ns	ns	ns	ns
S × N × (R vs. B)	ns	ns	*	***	***	ns	ns	ns	ns	ns	**	ns	ns	ns	ns	ns	ns	ns	ns

Significance of F: ns, not significant for P ≤ 0.05; *, **, and *** = significant for P ≤ 0.05, 0.01 and 0.001. Y, yield; DM, dry Matter; NC, nitrate concentration; NRA, nitrate reductase activity; Act, nitrate reductase activity actual; Tot, nitrate reductase activity total; AcSt, nitrate reductase activity activation state; GSLs, glucosinolates; 4HGB, 4-hydroxyglucobrassicin; 4MGB, 4-methoxyglucobrassicin; GBC, glucobrassicin; DMB, dimeric-glucosativin; GER, glucoerucin; GNPF, gluconapoleiferin; GRA, glucoraphanin; GSV, glucosativin; DGTB, diglucothiobeinin; PRO, progoitrin; GAL, glucoalyssin; GNT, gluconasturtiin; GSLs Tot, total of glucosinolates). B, BLUE, peak at 460 nm; R, RED, peak at 660 nm; R + B, RED + BLUE, 75% red + 25% blue.

## Results

### Yield and Dry Matter (DM) Percentage

Yield was not influenced by the species used, while a significant increase was produced by the nitrogen level, with 8 mM nitrogen treatment producing 200% more crop compared to the 1 mM treatment (Table 3). By comparing the yield obtained by the three LED spectra, RED light produced 31% more than B and R + B (Tables 2, 3).

**TABLE 3:** Average yield and dry matter of rocket as a function of light treatment, nitrogen level and species.

Treatment	Yield (g plant^–1^)	Dry matter (g⋅100 g^–1^ fresh weight)
**Species**		
*Eruca sativa*	104	9.66
*Diplotaxis tenuifolia*	114	8.21
**Nitrogen level (mM)**		
1	72	10.28
8	144	7.68
**Light**		
SL	114	8.95
BLUE	99	8.60
RED	129	9.45
R + B	98	8.62

*The variance results of the main treatments, interactions and contrasts are reported in Table 2.*

**FIGURE 1:**
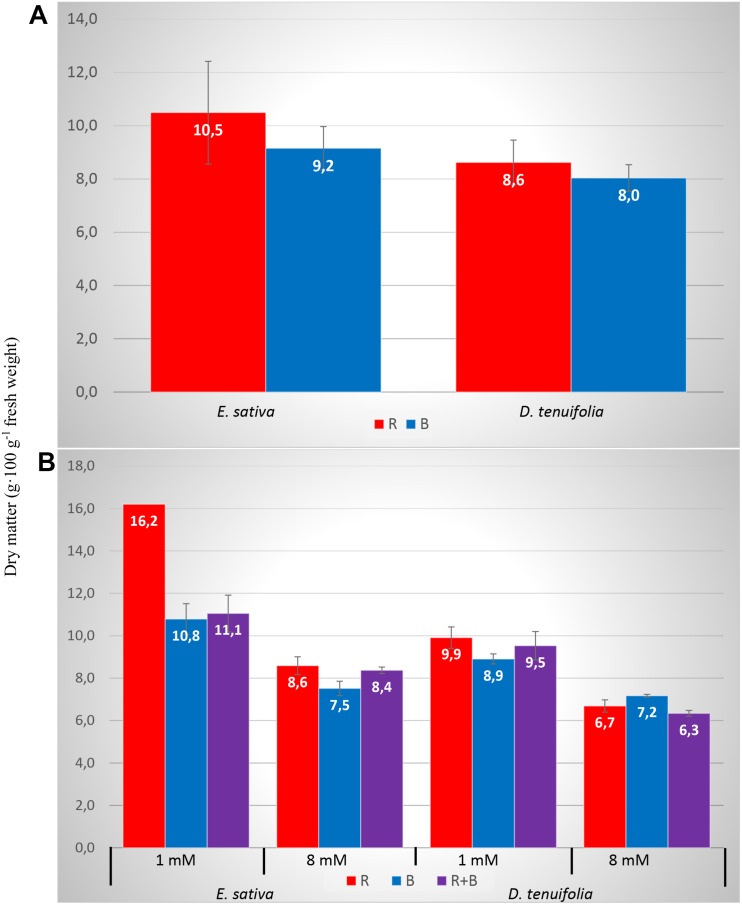
Dry matter percentage of rocket leaves as influenced by interactions between species and light **(A)** and species, nitrogen level and light **(B)**. Vertical bars represent the standard error.

The DM percentage was influenced by the species, by the N level, from their interaction, and from an interaction between some light spectra (Table 2). In general, the 1 mM treatment produced a significantly higher DM percentage with respect to 8 mM, and *Eruca* showed a higher percentage DM compared to *D. tenuifolia* (Table 3). The DM percentage was modulated by the light treatment, as the RED light increased the amount of DM with respect to BLUE, especially in *Eruca* compared to *Diplotaxis* (Figure 1A). Such interaction was further modulated by the N level, since *E. sativa* produced a higher content of DM under the RED treatment with the lower nitrogen level (Figure 1B).

### Nitrate Concentrations

**FIGURE 2:**
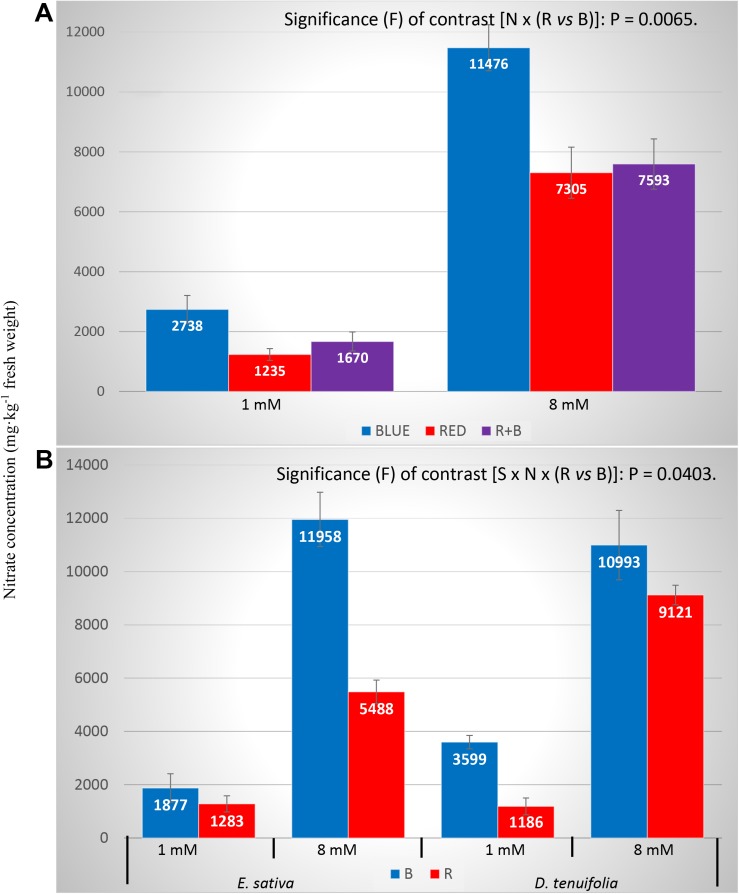
Nitrates concentration of rocket leaves as influenced by interaction light x nitrogen level **(A)** and species × nitrogen level × light **(B)**. Vertical bars represent the standard error. S, species (*E. sativa* and *D. tenuifolia*); N, nitrogen level into the nutrient solution (1 and 8 mM); B, BLUE, peak at 460 nm; R, RED, peak at 660 nm.

The R and R + B treatments produced the lowest concentrations, while BLUE light the highest, even when the nitrate content was greatly influenced by the nitrogen concentration in the NS. The higher the nitrogen treatment, the higher the nitrate concentration (Table 2 and Figure 2A), which is what would be expected. Yet, the influence of light and N level on nitrate concentrations was species dependent: in the BLUE treatment in *D. tenuifolia* (1 mM), the NO_3_^–^ concentration was threefold higher than that of RED (Figure 2B); while in *E. sativa* (with the same level of N), the nitrate content was increased only by 46% (Figure 2B). Such light-driven difference was present, in both species; also in the 8 mM treatments, and more pronounced in *E. sativa* (>200% increase) than in *D. tenuifolia* (Figure 2B).

### Nitrate Reductase Activity

As the behavior of NR_act_ and NR_total_ has followed the same pattern as each other (Table 2), only the data of the former will be commented on by referring to it as NR activity.

**FIGURE 3:**
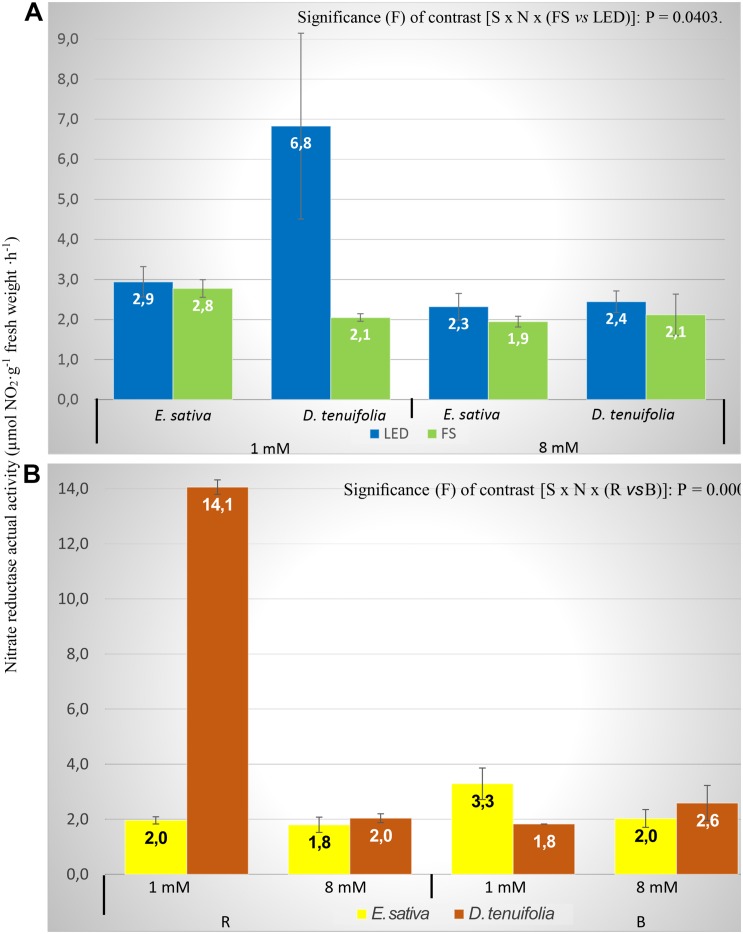
Nitrate reductase actual activity (NRact) of rocket leaves in function of interactions species × nitrogen level × light **(A)** and species x light spectrum **(B)**. Vertical bars represent the standard error. S, species (*E. sativa* and *D. tenuifolia*); N, nitrogen level into the nutrient solution (1 and 8 mM); B, BLUE, peak at 460 nm; R, RED.

The LED lights significantly increased the NR activity in comparison to the FS treatment with both N treatments, except for *E. sativa* (1 mM; Figure 3A), with values almost the same with LED or FS. *D. tenuifolia* had on average greater values with LED, as with 1 mM N the NR activity value was three fold higher in LED compared to FS (6.83 and 2.05 μmol NO_2_^–^⋅g FW^–1^⋅h^–1^, respectively; Figure 3A). From Table 2 is clear that the greater difference is between the RED and BLUE light treatments, which is consistent with observations from changing nitrate concentration. In Figure 3B the interaction between species, N level and RED and BLUE spectra is reported. For the 8 mM N level, *D. tenuifolia* showed slightly higher NR activity for both spectra (Figure 3B). With the lower N level, BLUE light caused a higher NR activity in *Eruca*, while in *Diplotaxis* RED light showed a NR activity that was eightfold higher than that of BLUE light (Figure 3B).

### Glucosinolates

**FIGURE 4:**
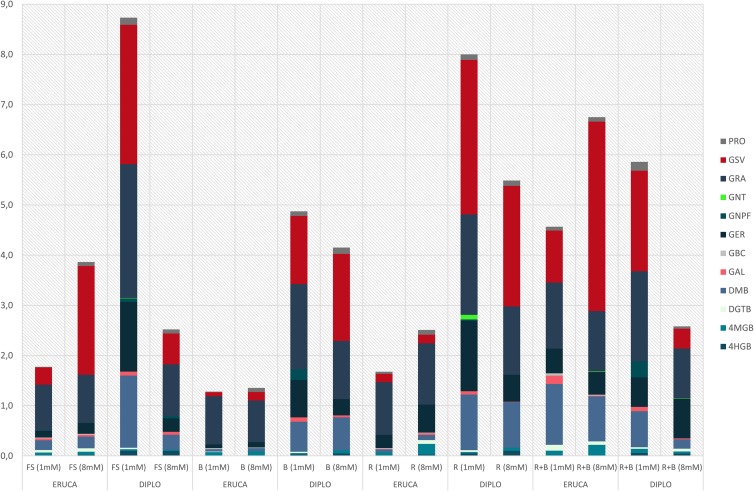
GLSs content of rocket leaves (*E. sativa* and *D. tenuifolia*) in function of light and N level. (ERUCA, *E. sativa*; D, *D. tenuifolia*); 1 and 8 mM, nitrogen level into the nutrient solution; FS, full spectrum; B, BLUE, peak at 460 nm; R, RED, peak at 660 nm; R + B, RED + BLUE, 75% red + 25% blue. 4HGB, 4-hydroxyglucobrassicin; 4MGB, 4-methoxyglucobrassicin; GBC, glucobrassicin; DMB, dimeric-glucosativin; GER, glucoerucin; GNPF, gluconapoleiferin; GRA, glucoraphanin; GSV, glucosativin; DGTB, diglucothiobeinin; PRO, progoitrin; GAL, glucoalyssin; GNT, gluconasturtiin.

Our results highlighted that the main discriminating factor in influencing the total GSLs concentrations were the species and their interactions with light treatment or nitrogen level (Table 2). The species influenced total GLSs content (Figure 4), but no significant effects on the accumulation of GSV, DGTB, GAL, GNT (refer to Table 2 for acronyms) were observed.

Light had different effect on total GLSs depending on the species. In *E. sativa* the blue light produced lower concentrations of total GLSs, but in *D. tenuifolia* this was not the case (Figure 4). Observing the values of the GLSs in the two species with the same N level, it is evident that in *E. sativa* the concentration of GSLs was always lower with respect to *D. tenuifolia*, with the exception of FS 8M and R + B 8mM treatments (Figure 4). The lower total GLSs content was observed under the BLUE light, while the highest value was produced under the R + B (1.28 and 6.75 mg⋅g DW^–1^, respectively – Figure 4). In *D. tenuifolia*, the BLUE light with 1 mM of N produced a total content of GLSs that was only 56 and 61% if compared to the correspondent values and N concentration of FS and RED light, respectively (Figure 4).

Taking into account the interaction between the light and the nitrogen level, *D. tenuifolia* showed in general a higher content than *Eruca*. In particular, in *Diplotaxis* with 1 mM N, the total GSL content was 3.4-folds higher than with 8 mM N – Figure 4) as opposed to *E. sativa*, in which the higher concentration was detected with 8 mM treatment (Figure 4).

## Discussion

### Yield and Dry Matter (DM) Percentage

The LED spectra showed a different behavior with respect to yield, as RED light produced a higher yield than B and R + B treatments (Tables 2, 3). This result agrees with those reported by Długosz-Grochowska et al. (2017) who found a higher yield in *Valerianella locusta* (L.) when red light was present, as red light is reported to be the most effective at activating photosynthesis (Kuno et al., 2017). In addition, Hogewoning et al. (2010) reported that an increases in the red light fraction are associated with a simultaneous decrease in stomatal conductance, which has an inverse relationship with WUE. Yet, highest instantaneous photosynthesis under monochromatic red light did not always lead to optimal growth on the long run for certain crops such as cucumber (Hogewoning et al., 2010) or ornamentals (Zheng and Van Labeke, 2017). Our results thus indicate that rocket tolerates monochromatic red light regimes; probably because it is a fast growing, short cycling crop.

Similarly to yield, the DM percentage was not influenced by light, but it was dependant on species, nitrogen level, and interaction with some LED spectra. More in detail, DM was higher with 1 mM level and in *Eruca*, but was modified by light treatment (Figures 1A,B). This result is due to the effect of the light on nitrate concentration in leaves. In fact, as reported by other studies on lettuce (Blom-Zandstra et al., 1988), the N level exerts an influence on the DM, since high nitrate concentrations derive from increased nitrogen availability, and is linked to a lower dry matter percentage. Nitrate accumulation causes an osmotic effect, which in turn decreases the dry matter content (Tei et al., 2000). The role of light spectra in increasing the DM percentage in leafy vegetables is still unclear: Lobiuc et al. (2017) have reported that DM of basil was significantly increased by an increasing intensity of blue light, while others (Ohashi-Kaneko et al., 2007; Wojciechowska et al., 2015) reported an increase of DM in spinach, komatsuna and lamb’s lettuce plants grown under red light. Response to light treatments is therefore likely to be highly species specific, and not universally applicable to all crops.

### Nitrate Concentrations

The experiment took place at a relative low photon flux density (150 μmol m^–2^ s^–1^) favoring the accumulation of nitrate as an osmolyte in the vacuoles. At this light intensity, light spectra had a significant influence on NO_3_^–^ concentration, and we observed some differences according with different spectra and N level; even if such differences were species dependant (Table 2 and Figures 2A,B).

Generally speaking, the concentration of NO_3_^–^ in leaves increases with increasing nitrogen content in the growing media (Santamaria et al., 1997), even if this aspect may be affected by other parameters, such as light spectrum, species, and nutrient solution management (Gonnella et al., 2004). It is subject to dynamic changes in function of genetic and environmental factors (Anjana and Iqbal, 2007). The light spectrum has a prominent role in determining the NO_3_^–^ concentration in leafy vegetables, and a lessening of nitrate concentration when supplementing plants with RED light. This has been reported by several authors in different species (Ohashi-Kaneko et al., 2007; Urbonavièiute et al., 2007; Wojciechowska et al., 2016; Długosz-Grochowska et al., 2017). Such reduction is in line with the observation that the RED component of the light, which is effectively adsorbed by the phytochrome, may stimulate NR activity (Lillo, 2004). It is noteworthy from commercial point of view that, with the higher N concentration in the NS, the RED light in *E. sativa* had a NO_3_^–^ concentration below the limits imposed by the Regulation (EC) N. 1258/2011 (5,488 mg⋅kg^–1^ of fresh weight), while in *D. tenuifolia* such limits were exceeded in both light treatments (Figure 2B). This is a fundamental finding, since the product with nitrate concentrations above the aforementioned limits cannot be legally sold on the market.

It is well known that genotypic variability influences nitrate concentrations (Anjana and Iqbal, 2007; Colonna et al., 2016), and that *D. tenuifolia* accumulates higher amounts than *E. sativa* (Santamaria et al., 2002). This difference may be explained by the difference in relative growth rate (RGR) between the two species. Ter Steege et al. (1999) reported that species with a high RGR [such as *E. sativa* – tends to have a faster growth rate as it is an annual species (Tripodi et al., 2017) – have a nitrate influx that may be 20–40% lower compared to species with lower RGR (in our case, *D. tenuifolia*, as it has a perennial behavior; Tripodi et al., 2017)]. However, nitrate accumulation in plants is a complex process, with main factors influencing nitrate accumulation that are mainly nutritional, environmental and physiological; as reported by Anjana and Iqbal (2009).

### Nitrate Reductase Activity

The NR activity (both for NR_act_ and NR_total_) was significantly influenced by all the factors considered, and their interactions (Table 2 and Figures 3A,B), whilst the activation state was not affected by any parameters (Table 2). This is not surprising, since the activation state of NR is not always correlated with total NR activity in leaves (Man et al., 1999). In general, LED lights increased the NR activity, with the exception of *E. sativa*, with 1 mM level of nitrogen (Figure 3A). The most remarkable difference was observed between RED and BLUE (Table 2) even if such difference was modulated by N concentration and species (Figure 3B). The importance of RED light in modulating the NR activity is well known, as the expression of NR is promoted by light absorbed by the phytochrome (Lillo, 2008). Therefore, the higher NR activity might be associated with the red spectrum that induces phytochrome phototransformations (Viršilë et al., 2018) that result in an increased synthesis of the enzyme, that is effectively mediated by phytochrome (Barro et al., 1989).

Typically, the activity of NR is driven by the concentration of nitrates in the substrate, so the higher their concentration, the higher the activity of NR. However, such activity is regulated by multiple parameters, such as light (including unconsidered spectra, such as green light wavelengths; Bian et al., 2018), temperature, salts, CO_2_, pH, and substances that regulate growth (Kaiser and Huber, 1994; MacKintosh and Meek, 2001; Garg, 2013; Yanagisawa, 2014; Schoenbeck et al., 2015). Other factors, apart from nitrate, are also involved in the control of synthesis and degradation of NR (Man et al., 1999). With regard to the nitrate concentration, Chen et al. (2004) found that by increasing concentration in the substrate, the NR activity changed only when passing from the lowest concentration to the following level, and then stopped. This may imply that a threshold of nitrate concentration exists in the metabolic pool that regulates nitrate reductase activity (Chen et al., 2004). Conversely, Man et al. (1999) observed that in plants grown with low nitrate content, the NR activity was only 30% of the NR activity of plants with high nitrate content. However, in the former, the activation state of NR during the dark phase was almost double than that observed in light, thus compensating for the lower value of NR activity during the day.

### Glucosinolates

The importance of GSLs and their concentrations in rocket is twofold: some compounds are responsible for the bitter taste and, secondly, the myrosinase breakdown products (mainly isothiocyanates; ITCs), have been demonstrated to be effective in preventing the risk of cardiovascular disease and some types of cancer (Bennett et al., 2007; Herr and Büchler, 2010; Melchini and Traka, 2010; Possenti et al., 2016), as well as producing characteristic pungency of Brassicaceae such as rocket (Bell et al., 2018).

The most important parameter that influenced GLS concentrations in our experiment was the species considered (Figure 4), but not for all individual glucosinolates (Table 2). However, the concentrations of total GSLs reported in the literature is highly variable; Pasini et al. (2012) and Di Gioia et al. (2018) found higher concentrations than those found in our experiments, while others Bell et al. (2015) reported total GLSs values closer to those found in the present experiment.

The species exerted an influence on the way the light spectra acted on GLSs concentration in the chosen cultivars: the blue light produced the lowest concentrations in *E. sativa*, but not in *D. tenuifolia* (Figure 4). Such results agree with Qian et al. (2016), who have observed in Chinese kale a decrease of total GSLs content when blue light was used. The final concentration of GSLs depends on not only the light treatment and the species, but also other parameters. For example, with respect to blue light, Kopsell and Sams (2013) found that blue light influenced positively total aliphatic GSLs concentrations. In our study no aliphatic GSL was influenced by the light alone (Table 2), but by an interaction between species and light (DMB and GSV) or species and N level (GNPF, GRA, GSV, and PRO – Table 2). The prominent role of the species in determining the GSL concentrations in our experiment may be inferred by observing the interactions between the experimental factors: where an interaction is present, the species always had a role, with the exception of 4MGB (Table 2).

It is noteworthy to mention that the concentration of total GSLs in rocket is not always unequivocally related to the species. In an experiment with 37 rocket salad accessions (both *E. vesicaria* and *D. tenuifolia*), Pasini et al. (2011) did not find any significant differences regarding the GSL content, while Di Gioia et al. (2018) reported a plant genotype influence. Variability is known to be high between individual accessions of each respective species (Bell et al., 2015) and so it is difficult to draw broad conclusions between the species based on these data alone. Such apparently inconsistent results are not unexpected, as GSL concentrations may vary as a function of several parameters, such as developmental stage, stress, plant age, photoperiod, temperature, and salinity (Hasegawa et al., 2000; Agerbirk et al., 2001; Coogan et al., 2001; Ahuja et al., 2010; Herr and Büchler, 2010) and geographical origin. These all have a significant effect on the profiles and concentrations of GSLs (Bennett et al., 2007). The crop environment exerts a great influence, i.e., if the cultivation has been done in field, controlled environment or with hydroponic systems (Bell et al., 2015) and it is well known that plant genotype and phenological stage of the final product (e.g., a completely developed leaf, sprouts or microgreens) all play important roles in determining the GSL content in plants (Padilla et al., 2007; Velasco et al., 2007; Wentzell and Kliebenstein, 2008; Francisco et al., 2011; Pérez-Balibrea et al., 2011; Agneta et al., 2014).

Nitrates and GSLs are key factors that determine the sensory and health-related quality of rocket crop and they are both localized in the plant vacuoles (Helmlinger et al., 1983). The concentration of GSLs depends also on the nitrogen form and concentration, since NH_4_^+^ can decrease their concentration, while NO_3_^–^ can significantly increase it (Kim and Ishii, 2006). The nitrogen level also influenced the total GLSs content jointly with light spectra: in *D. tenuifolia* RED and FS light, with 1 mM of nitrogen, produced a total GLSs content 1.6- and 1.8-fold higher, respectively, than BLUE light at same N level. Such effects have been previously observed by Omirou et al. (2012), who reported that the biosynthesis of GLSs is influenced by N levels; even with an observed significant interaction with the sulfur level. Indeed, in our experiments the S level into the nutrient solution was slightly higher in 1 mM with respect to 8 mM (76 vs. 29 mg⋅kg^–1^, respectively), as in the 1 mM treatment we added potassium in the form of K_2_SO_4_ – instead of KNO_3_, to reduce the N concentration to 1 mM into the nutrient solution. Such results agree with those reported by De Pascale et al. (2008), who reported that in Brassicaceae species both yield and quality are strongly dependent on the N:S ratio of the nutrient solution.

By considering the different classes of GSLs, aliphatics had a prominent role (Table 2), by representing 94.5 and 97.3% of the total for *E. sativa* and *D. tenuifolia*, respectively (Figure 4). In accordance with Omirou et al. (2012) who found that in *E. sativa*, the increase of the N supply, reduced most aliphatic GSLs and increased the indolic GSLs, in a similar manner to our findings. However, the influence of N on GSL concentrations is highly variable, and it has been reported that the increase of N level may reduce, increase, or have no effect on GSL concentration and composition, depending on the Brassicaceae species (Kim et al., 2002; Kopsell et al., 2007; Schonhof et al., 2007; Omirou et al., 2009).

## Conclusion

The use of artificial lights is becoming more and more important in vegetable production; in particular in those environments with reduced solar radiation, such as in greenhouses and in Northern countries; especially during the autumn and winter period. Among the leafy vegetables, rocket (both *E. sativa* and *D. tenuifolia*) contain some interesting compounds for the benefit of human health, but may also accumulate high concentrations of nitrates in the leaves. Our results indicate that under low light levels, red light is able to reduce the nitrate concentration in leaves; and in particular by increasing the nitrate reductase activity. This reduction has been observed to be species and nitrogen fertilization dependent. The yield and dry matter are influenced primarily by nitrogen level, although red light increased yields with respect to blue and red + blue, up to a level similar to that of full spectrum.

Considering the total content of GSLs, in *D. tenuifolia* the red component of the light alone (or the FS, but only with 1 mM of nitrogen) or mixed with blue (in *E. sativa*) increased their concentration. This indicates that each species may respond differently to light treatments and they should not be treated the same from a cultivation perspective. Such results are of interest to growers as they provide useful insights on the light spectra that should be used to improve the nutritional value of the rocket crop. Finally, the LED lights may be used in a real crop scenario, in both pre and post-harvest conditions, empowering growers and private companies to reduce nitrate concentration in rocket that may hamper the commercial sale of such product.

## Data Availability Statement

The datasets generated for this study are available on request to the corresponding author.

## Author Contributions

AS: substantial contributions to the conception or design of the work, drafting the work, final approval of the version, and nitrate reductase analysis. LB: glucosinolates analysis, interpretation of data, revised the article critically, and final approval of the version. PS: analysis and interpretation of data and final approval of the version. CW: glucosinolates analysis, interpretation of data, revised the article critically, and final approval of the version. M-CV: substantial contributions to the conception or design of the work, drafting the work, analysis and interpretation of data, and final approval of the version.

## Conflict of Interest

The authors declare that the research was conducted in the absence of any commercial or financial relationships that could be construed as a potential conflict of interest.
